# Obesity-related loci in *TMEM18*, *CDKAL1* and *FAIM2* are associated with obesity and type 2 diabetes in Chinese Han patients

**DOI:** 10.1186/s12881-020-00999-y

**Published:** 2020-03-30

**Authors:** Jing Kang, Ren-Chu Guan, Ying Zhao, Yan Chen

**Affiliations:** 1 Department of Endocrinology, The Second Hospital of Jilin University, No. 218 Ziqiang Street, Changchun, 130000 China; 2 College of Computer Science and Technology, Jilin University, Changchun, 130000 China

**Keywords:** SNP, Genetic variants, Obesity, Type 2 diabetes

## Abstract

**Background:**

Several obesity susceptibility loci in genes, including *GNPDA2*, *SH2B1*, *TMEM18*, *MTCH2*, *CDKAL1*, *FAIM2*, and *MC4R*, have been identified by genome-wide association studies. The purpose of this study was to investigate whether these loci are associated with the concurrence of obesity and type 2 diabetes in Chinese Han patients.

**Methods:**

Using the SNaPshot technique, we genotyped seven single nucleotide polymorphisms (SNPs) in 439 Chinese patients living in Northeast China who presented at The Second Hospital of Jilin University. We analyzed the associations between these seven alleles and clinical characteristics.

**Results:**

Risk alleles near *TMEM18* (rs6548238) were associated with increased waist circumference, waist/hip ratio, body mass index (BMI), fasting plasma glucose, hemoglobin A1c, diastolic blood pressure, triglycerides, total cholesterol, and low-density lipoprotein-cholesterol; risk alleles of *CDKAL1* (rs7754840) were associated with increased waist circumference and waist/hip ratio; and *FAIM2* (rs7138803) risk alleles were linked to increased BMI, diastolic blood pressure, and triglycerides (all *P* < 0.05). After adjusting for sex and age, loci near *TMEM18* (rs6548238) and *FAIM2* (rs7138803), but not *SH2B1* (rs7498665), near *GNPDA2* (rs10938397), *MTCH2* (rs10838738) and near *MC4R* (rs12970134), were associated with increased risk for type 2 diabetes in obese individuals.

**Conclusion:**

We found that loci near *TMEM18* (rs6548238), *CDKAL1* (rs7754840), and *FAIM2* (rs7138803) may be associated with obesity-related indicators, and loci near *TMEM18* (rs6548238) and *FAIM2* (rs7138803) may increase susceptibility of concurrent type 2 diabetes associated with obesity.

## Background

Type 2 diabetes, a common metabolic disease, is a global pandemic and has spread from developed countries to emerging economies, especially China [1]. Diabetes has become a challenging public health problem in China, affecting 98.4 million adults [2]. A combined effect of genetic and environmental risk factors contributes to the development of type 2 diabetes [3]. Like type 1 diabetes, type 2 diabetes is at least partially hereditary. Type 2 diabetes is considered a multigenic disorder, and therefore it is challenging to find diabetes susceptibility genes. Rapid advances in sequencing technology during the last 10 years have facilitated the discovery of genetic factors for type 2 diabetes. Single nucleotide polymorphisms (SNPs), common genetic variations, are associated with an increased risk of developing type 2 diabetes. So far, many type 2 diabetes susceptibility loci have been identified using genome-wide association (GWA) studies. Many of those genetic susceptibility loci are associated with beta-cell function and/or insulin sensitivity [4]. Type 2 diabetes has been recognized as a heterogeneous disorder, including obese and non-obese types. Therefore, obese and non-obese type 2 diabetic patients may have distinct genetic susceptibility loci.

Beyond genetic susceptibility, type 2 diabetes is closely related to lifestyle factors, such as obesity. Obesity results from excess body fat accumulation and is a major risk factor for type 2 diabetes. Obesity is partially due to environmental factors and lifestyle choices, but is also attributed to genetic susceptibility. To date, GWA analyses have identified more than thirty susceptibility loci robustly associated with obesity measured by body mass index (BMI). Those loci are in or near genes, including *GNPDA2*, *SH2B1*, *TMEM18*, *MTCH2*, *CDKAL1*, *FAIM2*, and *MC4R* [5–8]. It has been reported that the genotype-phenotype association varies in diverse groups of patients, and results need to be verified in a specific population [9]. Most of these obesity risk loci are associated with pattern and content of food intake. For example, obesity risk alleles in *MC4R* were shown to be associated with an increased caloric intake and a higher percentage of calories from fat [10]; *SH2B1* obesity risk alleles were linked to increased fat intake [11]. However, it is unknown whether these obesity susceptibility loci are associated with the development of type 2 diabetes in obese patients.

This study aimed to investigate the association of these seven obesity susceptibility loci with being overweight and obesity in the Chinese Han population living in Northeast China and whether these risk loci are associated with the concurrence of obesity and type 2 diabetes.

## Methods

### Study population

A total of 249 patients, who had been diagnosed with T2DM in the Department of Endocrinology at the Second Hospital of Jilin University during the period from October 2015 to October 2017, were enrolled in the study. Inclusion criteria included: (1) age 18–65 years; (2) compliance with standard treatment guidelines for T2DM, issued by the WHO diagnostic criteria in 1999 [12]. (3) BMI ≥ 24 kg/m^2^. Patients with ketosis during the last 6 months or impaired hepatic and renal function were excluded. Patients with type 1 diabetes, gestational diabetes, and other secondary diabetes were also excluded. According to the 2017 edition of the Chinese guidelines [13] for the prevention and treatment of type 2 diabetes, overweight and obesity were defined as 24 ≤ BMI < 28 kg/m^2^ and BMI ≥ 28 kg/m^2^, respectively. Using these criteria, the study included 126 patients with type 2 diabetes who were overweight (male: 72; age: 50.38 ± 14.47), 123 patients with type 2 diabetes and obesity (male: 93; age: 47.71 ± 16.28). And 190 healthy participants (male: 115; age: 44.24 ± 16.84) who had undergone physical examination during the same period. All subjects were genetically unrelated Han Chinese individuals living in Northeast China. The study protocol was approved by the Institutional Review Board of The Second Hospital of Jilin University. Written informed consent was obtained from every participant.

### Anthropometric and laboratory measurements

Anthropometric parameters, including height, body weight, waist circumference, hip circumference, and systolic and diastolic blood pressure, were measured according to the standard protocol, and BMI was calculated using the formula: weight / squared height (kg/m^2^). Peripheral venous blood samples were drawn from the subjects after 8 to 12 h fasting. Plasma glucose, insulin, hemoglobin A1c (HbA1c), triglycerides (TG), total cholesterol (TC), low-density lipoprotein cholesterol (LDL-C), and high-density lipoprotein cholesterol (HDL-C) were measured using an automated biochemical analyzer. Insulin resistance index (HOMA-IR) was calculated using the formula: fasting glucose (mmol/L) x fasting insulin (μU/L) / 22.5; insulin sensitivity index (HOMA-IS) was calculated using the formula: 22.5 / fasting glucose (mmol/L) x fasting insulin (μU/L).

### SNPs selection and genotyping

Seven SNPs, including rs10938397 (Near *GNPDA2*), rs7498665 (*SH2B1*), rs6548238 (Near *TMEM18*), rs10838738 (*MTCH2*), rs7754840 (*CDKAL1*), rs7138803 (*FAIM2*), and rs12970134 (Near *MC4R*) reported to be associated with obesity in GWA studies were selected for genotyping [5–8].

Genomic DNA was extracted from the leukocyte pellets of the subjects’ peripheral blood samples using the Blood Genomic DNA mini kit (CWBIO, Beijing, China) according to the manufacturer’s protocol. Genotyping was performed using the SNaPshot technology from the Beijing Genomics Institute (BGI, Beijing, China). Primer and probe sequences for each SNP are listed in Tables 1 and 2.

**Table 1:** PCR primers

Primers	Sequences
rs10938397-F	AGCCGGGCATGTAAACTG
rs10938397-R	TTGCCAAAGGACATAGCT
rs6548238-F	ATTTAGGTGCCTTTGATTG
rs6548238-R	GAGCCAGTTGTAGGGATG
rs7498665-F	ACAGGGTAGACTGCTGGTGA
rs7498665-R	TACCTGTGGCTGTTTCCG
rs10838738-F	AAGTAGACGGCGAGACAG
rs10838738-R	TCAGATTTCGGAAGGATG
rs7754840-F	GGGGAAGAAGTAGTAATG
rs7754840-R	AAGCTGCTCTGAACATAC
rs7138803-F	GCCCTTGATTTCCTTCTC
rs7138803-R	CCTCTGCCACCCACTAAC
rs12970134-F	AAAGGTGGCTTCTTAGATTGA
rs12970134-R	AGATAGGCAGTGTGGAGAC

**Table 2:** Single base extension primers

Primers	Sequences
rs10938397-YF2	CACACCAAAATGTTTTTACTTTACTTCTCATGGGA
rs6548238-YF2	AAGTCCACAGCTGGGAGCACAGGGA
rs7498665-YF3	CCCCCAGAGTTGCCCCCCCGCATCCCCATTGAAGAGGGACCCCCA
rs10838738-YF2	TAAAAACTGTTCCATTCCTAGGCAC
rs12970134-YF2	TATTTCGGTTCTAAGCAACAGATACTGATACTGACTCTTACCAAACAAAGCATGA
rs7138803-YR2	TATGATTCTATGAAATACTTTGCACAGCAGGGTGA
rs7754840-YF2	AAAATCAACTGCTTGCTGTTGGGGAAGAAGTAGTAATGTTGGAAA

### Statistical analysis

Continuous variables are expressed as mean ± standard error (SE). Student’s t-test and Wilcoxon signed-rank test were used for comparison between two groups in variables which were normally and not normally distributed, respectively. Categorical variables are expressed as frequencies (percentages), and comparisons were assessed using Chi-square test or Fisher’s exact test. The associations between the candidate SNPs and type 2 diabetes were analyzed using univariant logistic regressions. Multivariant logistic regressions were used to analyze the associations between the significant candidate SNPs and type 2 diabetes after adjusting for age and sex. All tests were two-tailed. A *P* value < 0.05 was considered statistically significant. All statistical analyses were performed using SPSS18.0 for windows (SPSS, Inc., Chicago, Illinois).

The sample size was calculated as follows: alpha was set as 0.05, beta was set as 0.20, OR was set as 2.0, P1 was set as 24% according to a previous report [14], and P0 was set as 12% according to a previous report [15]. Then the sample size was calculated using the following formula:

$$ N=\frac{\left[{Z}_a\times \sqrt{2\overline{P}\left(1-\overline{P}\right)+{Z}_{\beta }}\sqrt{P_1\left(1-{P}_1\right)+{P}_0\left(I-{P}_0\right)}\right]}{{\left({P}_1-{P}_0\right)}^2} $$

It came out that at least 124 participants were needed in healthy control and 247 were needed in T2DM group. Finally, 190 in healthy control and 249 in T2DM group were enrolled.

## Results

### Clinical characteristics

The general clinical characteristics of subjects in the healthy control, type 2 diabetes with overweight, and type 2 diabetes with obesity groups are presented in Table 3. The average age was similar among the three groups. The percentage of males was significantly higher in the obese diabetes group compared to the healthy control and overweight diabetes groups (both *P* < 0.05). Compared to healthy controls, type 2 diabetic patients with overweight or obesity had significantly increased waist circumference, waist/hip ratio, BMI, fasting plasma glucose, HbA1c, systolic and diastolic blood pressure, TG, TC, LDL-C, fasting plasma insulin, HOMA-IR, and HOMA-IS and decreased HDL-C (all *P* < 0.05). However, these parameters were not significantly different between overweight and obesity groups.

**Table 3:** Clinical characteristics of healthy controls and type 2 diabetic patients with overweight or obesity

Parameters	Healthy controls (*n* = 190)	Overweight diabetes (*n* = 126)	Obese diabetes (*n* = 123)	Z/χ^2^	*P*
Age	44.24 ± 16.84	50.38 ± 14.47	47.71 ± 16.28	1.50	0.227
Male/Female	115/75	72/54	90/30^*#^	6.68	0.036
Waist circumference (cm)	84.24 ± 14.71	97.79 ± 15.90^*^	100.07 ± 14.88^*^	12.41	<0.001
Waist/hip ratio	0.89 ± 0.34	1.13 ± 0.33^*^	1.18 ± 0.26^*^	8.19	<0.001
BMI (kg/m^2^)	22.44 ± 4.50	27.07 ± 4.23^*^	28.30 ± 4.51^*^	18.64	<0.001
FPG (mmol/l)	7.33 ± 2.67	11.21 ± 3.69^*^	11.53 ± 3.69^*^	18.42	<0.001
HbA1c (%)	7.83 ± 3.16	11.29 ± 3.77^*^	10.18 ± 3.40^*^	10.32	<0.001
SBP (mmHg)	113.61 ± 13.65	137.10 ± 22.73^*^	137.76 ± 20.03^*^	19.78	<0.001
DBP (mmHg)	75.94 ± 9.57	86.64 ± 10.72^*^	86.76 ± 9.00^*^	15.67	<0.001
TG (mmol/L)	2.70 ± 1.58	5.48 ± 4.81^*^	5.18 ± 4.52^*^	5.77	0.004
TC (mmol/L)	3.80 ± 1.75	5.81 ± 2.38^*^	6.04 ± 2.22^*^	12.88	<0.001
HDL-C (mmol/L)	1.31 ± 0.66	0.85 ± 0.34^*^	0.93 ± 0.39^*^	10.48	<0.001
LDL-C (mmol/L)	2.36 ± 0.88	3.08 ± 0.98^*^	2.99 ± 0.90^*^	6.99	<0.001
FINS (μIU/mL)	12.45 ± 6.95	24.80 ± 19.79^*^	23.89 ± 12.23^*^	9.19	<0.001
HOMA-IR	4.66 ± 3.67	11.93 ± 9.45^*^	11.36 ± 7.27^*^	11.85	<0.001
HOMA-IS	0.01 (0.00, 0.02)	0.013 (0.01, 0.02) ^*^	0.012 (0.00, 0.02)	10.39	0.006

*FPG* fasting plasma glucose, *FINS* fasting insulin. * *P* < 0.05 vs. healthy controls; ^#^*P* < 0.05 vs. overweight diabetes

### Genotype distribution

The genotypes of these seven loci in healthy controls and type 2 diabetic patients with overweight and obesity are shown in Table 4. At the locus near *GNPDA2* (rs10938397), the AA genotype was most abundant in patients with overweight and type 2 diabetes (50.0%), and GG was most abundant in healthy controls (21.1%). At the *SH2B1* (rs7498665) locus, the AA genotype was most abundant in patients with overweight and type 2 diabetes (42.9%), and GG was most abundant in obese diabetic patients (24.4%). At the locus near *TMEM18* (rs6548238), the CC genotype was most abundant in patients with overweight and type 2 diabetes (76.2%), and TT was most abundant in healthy controls (60.5%). At the *MTCH2* (rs10838738) locus, both AA (57.1%) and GG (19.1%) genotypes were most abundant in patients with overweight and type 2 diabetes. At the *FAIM2* (rs7138803) locus, the AA genotype was most abundant in patients with overweight and type 2 diabetes (14.3%), and TT was most abundant in healthy controls (76.3%). At the *CDKAL1* (rs7754840) and the near *MC4R* (rs12970134) loci, the genotype distribution was not significantly different among these three groups.

**Table 4:** Genotypes of seven genetic loci

SNP	Locus	Genotype	Healthy controls n (%)	Overweight diabetes n (%)	Obesity diabetes n (%)	χ^2^	*P*
rs10938397	Near GNPDA2	AA	85 (44.7)	63 (50.0)	54 (43.9)	4.45	< 0.001
		AG	65 (34.2)	45 (35.7)	51 (41.5)		
		GG	40 (21.1)	18 (14.3)	18 (14.6)		
rs7498665	SH2B1	AA	75 (39.5)	54 (42.9)	30 (24.4)	24.73	< 0.001
		AG	70 (36.8)	63 (50.0)	63 (51.2)		
		GG	45 (23.7)	9 (7.1)	30 (24.4)		
rs6548238	Near TMEM18	TT	115 (60.5)	18 (14.3)	57 (46.3)	70.53	< 0.001
		CT	5 (2.6)	12 (9.5)	12 (9.8)		
		CC	70 (36.8)	96 (76.2)	54 (43.9)		
rs10838738	MTCH2	AA	95 (50.0)	72 (57.1)	48 (39.0)	37.74	< 0.001
		AG	90 (47.4)	30 (23.8)	57 (46.3)		
		GG	5 (2.6)	24 (19.1)	18 (14.6)		
rs7754840	CDKAL1	CC	45 (23.7)	27 (21.5)	21 (17.1)	6.73	0.15
		CG	65 (34.2)	57 (45.2)	57 (46.3)		
		GG	80 (42.1)	42 (33.3)	45 (36.6)		
rs7138803	FAIM2	GG	145 (76.3)	66 (52.4)	63 (51.2)	32.16	< 0.001
		AG	40 (21.1)	42 (33.3)	45 (36.6)		
		AA	5 (2.6)	18 (14.3)	15 (12.2)		
rs12970134	Near MC4R	GG	105 (55.3)	81 (64.3)	75 (61.0)	7.58	0.108
		AG	60 (31.6)	39 (31.0)	39 (31.7)		
		AA	25 (13.2)	6 (4.8)	9 (7.3)		

### Association between genotype and clinical characteristics

At the near *GNPDA2* (rs10938397), *SH2B1* (rs7498665), and *MTCH2* (rs10838738) loci, there were no significant differences in clinical characteristics between genotypes AA and GG + AG. At the locus near *MC4R* (rs12970134), clinical characteristics were similar between genotypes GG and GG + AG. As shown in Table 5, compared with patients harboring TT at the locus near *TMEM18* (rs6548238), patients with CC + CT genotypes had increased waist circumference, waist/hip ratio, BMI, fasting plasma glucose, HbA1c, diastolic blood pressure, TG, TC, and LDL-C, and decreased HOMA-IS and HDL-C (all *P* < 0.05). Patients with CC + CG at the *CDKAL1* (rs7754840) locus had higher waist circumference and waist/hip ratio compared to patients with the GG genotype (both *P* < 0.05). At the *FAIM2* (rs7138803) locus, patients with AA + AG genotypes had increased BMI, fasting plasma glucose, diastolic blood pressure, TG, and fasting plasma insulin compared to patients harboring GG (all *P* < 0.05).

**Table 5:** Comparisons of clinical characteristics among genotypes rs7754840, rs7138803, and rs6548238

	rs7754840		*P*	rs7138803		*P*	rs6548238		
	CC + CG	GG		AA+AG	GG		CC + CT	TT	*P*
Waistcircumference (cm)	96.68 ± 17.31	90.29 ± 14.56	0.040	97.11 ± 16.42	92.53 ± 16.53	0.139	97.27 ± 16.84	90.75 ± 15.66	0.030
Waist/hip ratio	1.11 ± 0.36	0.97 ± 0.25	0.027	1.09 ± 0.30	1.04 ± 0.35	0.345	1.15 ± 0.33	0.95 ± 0.29	0.001
BMI (kg/m^2^)	26.48 ± 4.98	25.98 ± 5.80	0.612	27.94 ± 4.94	25.25 ± 5.25	0.006	26.94 ± 4.57	22.52 ± 5.98	0.023
FPG (mmol/L)	10.07 ± 4.00	10.14 ± 3.67	0.925	10.97 ± 3.77	9.54 ± 3.85	0.046	11.01 ± 3.80	9.01 ± 3.69	0.004
HbA1c, %	9.90 ± 3.94	9.72 ± 3.36	0.799	10.30 ± 3.74	9.53 ± 3.70	0.268	10.70 ± 4.02	8.78 ± 3.05	0.004
SBP (mm Hg)	132.91 ± 23.98	124.93 ± 17.85	0.039	130.00 (112.00,115.00)	122.00 (110.00,150.00)	0.413	131.39 ± 22.03	128.20 ± 22.39	0.432
DBP (mm Hg)	84.03 ± 11.14	82.13 ± 10.60	0.360	86.45 ± 9.91	81.34 ± 11.16	0.012	85.91 ± 9.91	80.22 ± 11.39	0.004
TG (mmol/L)	4.61 ± 4.38	4.32 ± 3.69	0.705	3.66 (1.98,7.13)	2.62 (1.42,4.84)	0.041	5.46 ± 4.80	3.36 ± 2.76	0.005
TC (mmol/L)	5.39 ± 2.60	5.38 ± 2.60	0.44	5.38 ± 2.60	4.93 ± 2.38	0.324	5.88 ± 2.45	4.51 ± 1.99	0.001
HDL-C (mmol/L)	1.05 ± 0.54	0.98 ± 0.47	0.455	0.92 (0.58,1.06)	0.97 (0.70,1.22)	0.066	0.85 ± 0.36	1.23 ± 0.59	0.001
LDL-C (mmol/L)	2.92 ± 1.01	2.37 ± 0.87	0.176	2.99 ± 0.93	2.72 ± 0.98	0.133	2.98 ± 1.04	2.63 ± 0.85	0.050
FINS (μIU/mL)	22.27 ± 17.33	17.82 ± 10.06	0.076	19.60 (16.56,26.72)	13.18 (8.72,26.10)	0.007	21.50 ± 12.51	19.55 ± 17.88	0.484
HOMA-IR	6.81 (2.98,14.05)	5.60 (3.59,13.86)	0.677	10.46 (3.72,15.07)	5.35 (2.90,12.68)	0.083	10.01 ± 8.52	8.79 ± 7.31	0.399
HOMA-IS	0.010 (0.000,0.020)	0.010 (0.005,0.017)	0.184	0.012 (0.010,0.020)	0.010 (0.000,0.020)	0.662	0.010 (0.000,0.010)	0.016 (0.010,0.020)	0.001

Univariate logistic regression analysis showed that the loci near *TMEM18* (rs6548238) and *FAIM2* (rs7138803) were associated with type 2 diabetes. At the locus near *TMEM18* (rs6548238), subjects with CC + CT genotypes had an increased risk of diabetes compared to the TT genotype (odds ratio (OR) = 2.44, 95% confidence interval (CI): 1.11–5.37 *P* = 0.026). At the *FAIM2* (rs7138803) locus, individuals with AA + AG genotype had a higher diabetes risk compared to those with the GG genotype (OR = 2.72, 95% CI: 1.15–6.45, *P* = 0.023). However, the loci near *GNPDA2* (rs10938397), *SH2B1* (rs7498665), *MTCH2* (rs10838738), *CDKAL1* (rs7754840), and near *MC4R* (rs12970134) were not significantly associated with the concurrence of type 2 diabetes (all *P* > 0.05, Table 6).

**Table 6:** Univariant logistic analysis of the association of seven loci with type 2 diabetes

Loci	*b*	SE	*P*	OR	95% CI	
					Lower	Upper
rs10938397	−0.09	0.39	0.818	0.91	0.42	1.97
rs7498665	0.25	0.41	0.54	1.28	0.58	2.83
rs6548238	0.89	0.40	0.026	2.44	1.11	5.37
rs10838738	0.07	0.39	0.854	1.08	0.499	2.32
rs7754840	0.30	0.40	0.45	1.35	0.62	2.97
rs7138803	1.00	0.44	0.023	2.72	1.15	6.45
rs12970134	0.31	0.40	0.441	1.36	0.62	2.96

The loci that were significantly associated with diabetes risk in univariate logistic analysis were used in a multivariant regression analysis model to further study their association with type 2 diabetes. After adjusting for age and sex, the loci near *TMEM18* (rs6548238) and *FAIM2* (rs7138803) were significantly associated with diabetes (Table 7). Compared to the TT genotype, the subjects with CC + CT genotypes of rs6548238 had an increased diabetes risk with an OR of 2.27 (95% CI: 1.00–5.13, *P* = 0.05). For the *FAIM2* (rs7138803) locus, subjects with AA + AG had an elevated risk for type 2 diabetes compared to the GG genotype (OR = 2.67, 95% CI: 1.09–6.59, *P* = 0.033).

**Table 7:** Multivariant logistic analysis of the association between genetic loci with diabetes risk

	*b*	SE	*P*	OR	95% CI	
					Lower	Upper
Sex	−0.309	0.44	0.487	0.74	0.31	1.75
Age	−0.43	0.43	0.314	0.647	0.28	1.51
rs7138803	0.98	0.46	0.033	2.67	1.09	6.59
rs6548238	0.82	0.42	0.05	2.27	1.00	5.13
Constant	0.68	0.64	0.29	1.97		

## Discussion

Many obesity susceptibility loci identified from large-scale GWA studies have been confirmed in specific populations. This study investigated the association of seven common obesity risk loci with overweight/obesity and type 2 diabetes in a Chinese Han population. We found that the loci near *TMEM18* (rs6548238) and *CDKAL1* (rs7754840) were associated with increased waist circumference and waist/hip ratio, and *FAIM2* (rs7138803) was associated with increased BMI, diastolic blood pressure, and TG. After adjusting for age and sex, CT or CC genotypes at the locus near *TMEM18* (rs6548238) and AA or AG genotypes of the *FAIM2* (rs7138803) locus were associated with type 2 diabetes. In addition, these association relationships were dependent on BMI.

In this study, we found that CC or CT genotypes at the locus near *TMEM18* (rs6548238) were most frequent in overweight/obese patients with type 2 diabetes. Allele C was most common in overweight/obese diabetic patients, and CC or CT genotypes were associated with an increased diabetes risk, indicating that allele C may be a risk factor for concurrent obesity and diabetes. In addition, subjects with CC or CT genotypes of rs6548238 had elevated waist circumference, waist/hip ratio, BMI, fasting plasma glucose, HbA1c, TG, and TC, but declined HDL-C, suggesting that the locus near *TMEM18* (rs6548238) may increase obesity/diabetes risk through inducing metabolic disorders. In 2009, the Genetic Investigation of Anthropometric Traits Consortium conducted a large-scale meta-analysis involving more than 32,000 European subjects and found that the locus near *TMEM18* (rs6548238) was associated with obesity, with allele C being more important than allele T [16]. This result is consistent with the finding in our population. TMEM18 is widely expressed in several regions of the brain and is particularly abundant in the hypothalamus [17]. It was reported that TMEM18 increases mouse body weight and white/brown fat mass through increasing high-fat food intake [18]. In 2009, Willer et al. first reported that SNPs near *TMEM18* were linked to obesity in humans and that the locus near *TMEM18* (rs6548238) was associated with increased BMI [16]. This finding was then confirmed in obese adults and children [19–23]. The CT or CC genotypes in the locus near *TMEM18* (rs6548238) were not associated with plasma insulin levels. Therefore, we speculated that the locus near *TMEM18* (rs6548238) may increase diabetes risk via inducing obesity and insulin resistance. An experimental study in *Drosophila* found that TMEM18 affected substrate levels via insulin and glucagon signaling [24]. Further functional studies are needed to understand the mechanisms by which the locus near *TMEM18* (rs6548238) regulates the development of obesity and type 2 diabetes.

Our study found that AA or AG genotypes of *FAIM2* (rs7138803) were most frequent in overweight/obese patients with type 2 diabetes in a Chinese Han population. Allele A was most common in overweight/obese diabetic patients, and AA or AG genotypes were associated with an increased risk for type 2 diabetes, indicating that allele A at this locus may be a risk factor for obesity and diabetes. Moreover, the subjects with AA or AG genotypes had increased BMI, fasting plasma glucose, diastolic blood pressure, TG, and fasting plasma insulin compared to those with the AA genotype. A previous large-scale GWA study identified *FAIM2* (rs7138803) as being associated with obesity in Caucasian adults [18]. This result was then confirmed in an Asian population [25], where this association was absent in two Chinese populations from Sichuan and Beijing [26, 27]. These inconsistent findings might be due to differences in ethnicity and sample size. It was reported that FAIM2 is highly expressed in the hippocampus and may be involved in the development of appetite controlling nerves and apoptosis of adipocytes [28]. Therefore, polymorphisms in *FAIM2* (rs7138803) are speculated to induce obesity by enhancing appetite and suppressing adipocyte apoptosis, which are consistent with the current study showing an association of risk allele and increased BMI. In this case, the increased risk for diabetes is likely due to obesity. The risk allele of *FAIM2* (rs7138803) was associated with increased diastolic blood pressure, which might be secondary to obesity.

Previous studies in European and Asian populations found that *CDKAL1* is associated with type 2 diabetes [29–31], and the C allele of rs7754840 was associated with the highest risk for diabetes [25]. Although the function of CDKAL1 is not fully understood, the CDKAL1 protein likely functions similarly to cyclin dependent kinase 5 (CDK5). A recent in vitro study demonstrated that CDK5 caused functional impairment of islet beta cells [32]. In addition, knockout of *Cdkal1* in mice impaired first-phase insulin exocytosis in beta cells, likely through regulating K_ATP_ channel responsiveness [33]. A study including Finnish men demonstrated that *CDKAL1* (rs7754840) was associated with increased risk for type 2 diabetes, likely via impairing insulin secretion [34]. The current study did not show an association between *CDKAL1* (rs7754840) and diabetes, which may be due a to relatively small sample size. We found that subjects with CC or CG genotypes at the *CDKAL1* (rs7754840) locus had increased waist circumstance and waist/hip ratio, indicating that rs7754840 may increase diabetes risk as well as abdominal obesity. This association needs to be further investigated in larger sample-sized studies.

Associations of the loci *SH2B1* (rs7498665), near *GNPDA2* (rs10938397), *MTCH2* (rs10838738), and near *MC4R* (rs12970134) with obesity have been shown in many studies [25, 35, 36]. Morris et al. found that *SH2B1* is involved in regulating diabetes risk by affecting insulin sensitivity [37]. The locus near *GNPDA2* (rs10938397) was associated with increased risk for obesity, which was independent of BMI [38]. A Chinese population study revealed that *MC4R* (rs12970134) was associated with type 2 diabetes after adjusting for BMI [39]. However, our study did not show that these four loci were associated with obesity and diabetes, which may be due to different ethnic populations. This study included Chinese Han population in Northeast China. In the follow-up work, we will conduct a larger sample size test for financing studies.

## Conclusions

Taken together, among seven common obesity risk loci, the loci near *TMEM18* (rs6548238), *CDKAL1* (rs7754840), and *FAIM2* (rs7138803) are associated with obesity, and loci near *TMEM18* (rs6548238) and *FAIM2* (rs7138803) are susceptibility loci for obese type 2 diabetes.

## Data Availability

The datasets generated and/or analysed during the current study are available in the NCBI ClinVar repository, ClinVar accession number: SCV001134937, SCV001160743, SCV001160724.

## Abbreviations

SNPs: Single nucleotide polymorphisms
GWA: Genome-wide association
HbA1c: Hemoglobin A1c
TG: Triglycerides
TC: Total cholesterol
LDL-C: Low-density lipoprotein cholesterol
HDL-C: High-density lipoprotein cholesterol
